# A Holiday Sermon

**Published:** 1887-09-10

**Authors:** 


### The Doctor's Pulpit.

### A HOLIDAY SERMON.

People who like to see work effectively done remember with admiration certain passages in *Cæsar's Commentaries.* The Roman legions showed the world of two thousand years ago how battles were won and countries subdued and ruled. The soldiers had discipline, energy, and pluck; the generals method and promptitude. We express no agreement with Cæsar's ambitions and projects: there is neither need to do so now, nor advantage in doing it. He lived, and thought, and fought and conquered, and his example is worthy of consideration as an example of efficiency. Work done, done thoroughly, and as well as it can be done, is always admirable.

Large numbers of modern Englishmen excite our admiration as much as Cæsar and his legions, and for similar reasons. They lay their plans—plans for broad, far-reaching, and prolonged operations; and they pursue them with a tenacity, an energy, an intelligence, and a determination which no examples in history have ever surpassed. But there is this difference between the English workers and the Roman legions, that the latter formed mechanical masses moved by a few inspiring spirits, whilst the former consist of innumerable individual workers, each making and pursuing his own plans to their natural termination. The Roman legion consisted of one man, conducting a many-headed machine. The English army of peaceful occupations is not mechanical, but individual; not one Cæsar with many legions, but many Cæsars, each one of them his own legion. We greatly admire our fellow-countrymen as a whole. In the Church, at the Bar, in politics, in commerce, in medicine, in science, in engineering, and in ploughing, mowing, hedging, and ditching, we are a capable people. We know it, we feel it, we are even inclined to be arrogant about it. Our descendants and cousins across the Atlantic who do not experience the restraints of age as we do, are unable to repress this consciousness of power which they inherit from us. They know they are able to "lick creation," and they say so. We don't say so, but like the parrot who wouldn't talk, we think it all the more.

Nobody will deny that we are a race of workers. But we want to continue to be workers, to continue not for ten years, but for a lifetime, not only in ourselves, but in our children and in our grandchildren. Does the reader "pooh, pooh" that? Let him pause a few moments and think the matter over again: he will conclude that we want to continue. But if we are to continue we must play as well as work. We are an absolutely foolish people for giving up play when we leave school. The man who works nine hours a day and plays three, will do much more and much better work all the year round than he who sticks to the desk or the counter the whole twelve. If we had our way we should surprise some people by the sweeping compulsory closing acts we should pass and enforce. The shopkeepers and all that class of people are utterly wrong-headed in keeping open their places of business in the way they do. Competition truly, we admit; but people must have goods; and if the shopkeepers had sense to understand their own interests they would take care that those goods should be purchased within reasonable hours.

Shopkeepers, however, blame other people; many people who don't take holidays have nobody to blame but themselves. But people who need and intend to continue their work to a reasonable age *must* take holidays. Holidays are better than physic, better than social advancement, better than wealth. They are absolutely indispensable. People will say, "they cannot spare the time"; "the claims of business are too urgent"; "the family necessities are too great"; and so on. Some foolish men and women who pride themselves on their success and popularity will tell us "it is better to wear out than to rust out." One such man we knew who always had this sentence upon his lips. Originally a robust man, capable of enduring an almost incredible amount of fatigue, he worked year after year until his brain was enfeebled, his appetite gone, and stimulants had become indispensable. Then the descent of Avernus was easy; he went down and down, until, at a little over fifty, he became paralysed, imbecile, and useless: a wreck: dead, and yet living: living, and yet dead. He had a large family, several of whom were quite unable to provide for themselves; and the disaster to the household was complete. Would it not have been much better that his prosperity and social advancement should have been more gradual; that his necessary expenses should have been kept at a lower figure, and that his health should have been maintained by adequate holidays?

What is an adequate holiday? Is it a fortnight, or three weeks, or a month, or two months, or six months? It is just so many weeks or months as a man requires to restore him to that condition of health and efficiency which is natural to him, and to give him a little overplus of strength for storage. The need for holidays will usually be in proportion to the capacity for work and the quantity of work done. Given a great working capacity with a long exercise of it, and the holidays must be complete and of proportionate length. A man who comes out with a mere pass degree may be restored to sound health by a cricket-match or a rat-hunt, but a senior wrangler would be all the better for a six months' yachting expedition. The bank clerk will probably perfectly recover his fitness for his sober and routine duties in a fortnight; the Government clerk perhaps by a visit to Kew and "tea and shrimps for ninepence"; but the stockbroker, the busy lawyer, the hard-worked doctor—especially if he be a general practitioner—should never take less than a month in the year, and two months if he can possibly get them. Of course in his absence the fees will fall off, and the *locum tenens* perhaps get into all manner of scrapes; but that must be risked. "Physician, heal thyself," is an excellent oracle, which should be painted in large letters over the door of every doctor's consulting-room.

What the poor Englishman is to *do* in his holidays the occupant of the Doctor's Pulpit is almost nonplussed to say. We are so terribly sober and dignified—shall we say dull-witted?—that we do not seem to be able to spend a holiday easily, cheerfully, economically, and successfully. We must found a Society for the discussion of the first principles of holiday-making. Then perhaps it will really be discovered what is the very best way for John Bull and his family to enjoy a few weeks of absolutely essential rest and change. In any case a holiday every year every one of us must have. It is one of the indispensable requisites of successful and continuous work; one of the most important resources of our high and progressive civilisation. That man shall wear the laurel leaf who best succeeds in prolonging our holidays and in making us thoroughly enjoy them.

(*To be continued.*)

